# Calibration and Validation of Simulation Parameters for Maize Straw Based on Discrete Element Method and Genetic Algorithm–Backpropagation

**DOI:** 10.3390/s24165217

**Published:** 2024-08-12

**Authors:** Fandi Zeng, Hongwei Diao, Yinzeng Liu, Dong Ji, Meiling Dou, Ji Cui, Zhihuan Zhao

**Affiliations:** 1 College of Mechanical and Electronic Engineering, Shandong Agriculture and Engineering University, Jinan 250100, China; zfd19508@163.com (F.Z.); dhw_0823@163.com (H.D.); lyz19971024@163.com (Y.L.); 13325259481@163.com (D.J.); meiling0508@163.com (M.D.); 2 College of Mechanical and Electrical Engineering, Inner Mongolia Agricultural University, Hohhot 010018, China; cuijiemail@163.com

**Keywords:** DEM, neural network, GA–BP, maize straw, peak compression force

## Abstract

There is a significant difference between the simulation effect and the actual effect in the design process of maize straw-breaking equipment due to the lack of accurate simulation model parameters in the breaking and processing of maize straw. This article used a combination of physical experiments, virtual simulation, and machine learning to calibrate the simulation parameters of maize straw. A bimodal-distribution discrete element model of maize straw was established based on the intrinsic and contact parameters measured via physical experiments. The significance analysis of the simulation parameters was conducted via the Plackett–Burman experiment. The Poisson ratio, shear modulus, and normal stiffness of the maize straw significantly impacted the peak compression force of the maize straw and steel plate. The steepest-climb test was carried out for the significance parameter, and the relative error between the peak compression force in the simulation test and the peak compression force in the physical test was used as the evaluation index. It was found that the optimal range intervals for the Poisson ratio, shear modulus, and normal stiffness of the maize straw were 0.32–0.36, 1.24 × 10^8^–1.72 × 10^8^ Pa, and 5.9 × 10^6^–6.7 × 10^6^ N/m^3^, respectively. Using the experimental data of the central composite design as the dataset, a GA–BP neural network prediction model for the peak compression force of maize straw was established, analyzed, and evaluated. The GA–BP prediction model’s accuracy was verified via experiments. It was found that the ideal combination of parameters was a Poisson ratio of 0.357, a shear modulus of 1.511 × 10^8^ Pa, and a normal stiffness of 6.285 × 10^6^ N/m^3^ for the maize straw. The results provide a basis for analyzing the damage mechanism of maize straw during the grinding process.

## 1. Introduction

Maize straw is an important renewable resource and feed source for animal husbandry. With the development of the agricultural circular economy, the recycling of agricultural solid waste has become an important research direction. The efficient utilization of crop straw is the key issue in this regard [1,2,3]. The deep processing and utilization of maize straw can alleviate the supply pressure of feed and reduce the residue of agricultural solid waste. It is also significant for promoting the development of biomass energy [4,5,6]. Using the discrete element method to establish a maize straw simulation model and carrying out parameter optimization design of the mechanism can improve straw productivity and utilization rates and promote the rapid development of straw-processing machinery.

Some researchers have carried out multiple studies on the calibration of the simulation parameters of agricultural materials. It is necessary to calibrate the relevant input parameters to establish an accurate discrete element parameter model and obtain a better direct interaction relationship between the material and the contact material [7,8,9]. Ma et al. [10] established a calibration model of mixed parameters for red clover seed and coated powder using the JCR model and verified the accuracy of the model parameters via experiments. Zhang et al. [11] constructed a calibration model for the mixed parameters of a maize root system and soil, providing a reference for the discrete element simulation of maize no-tillage operations. Jung et al. [12] established a simulation model for the flow parameters of soybeans with different pore sizes and water content and predicted the flow performance of soybeans with different water content via experiments. Zhang et al. [13] analyzed the relationship between different structures and bonding parameters of banana straw and established a high-precision discrete element model of banana straw. Zhao et al. [14] established a discrete element model of cotton straw compression and experimentally verified that the established model could accurately simulate the compression process of cotton straw. Tang et al. [15] studied the mechanical properties of rice straws of different lengths under vibration and non-vibration compression conditions, providing basic data for the development of straw compression machinery. Shi et al. [16] established a discrete element model of a wheat straw monomer and conducted physical and simulation experiments on the properties of wheat straw, such as tensile strength, compression, three-point bending, and shear stress. Zheng et al. [17] carried out discrete element parameter calibration for the cutting process of corn stalks. Traditional machine learning models require numerous datasets to be analyzed to obtain satisfactory results. The backpropagation (BP) neural network model can use less data to build the model [18,19]. Many scholars have studied the genetic algorithm (GA)–BP algorithm. Li et al. [20] compared the photovoltaic power generation prediction model constructed using three neural network algorithms, BP, GA–BP, and PSO (particle swarm optimization)–BP (backpropagation), and improved the prediction accuracy of photovoltaic power generation. Wei et al. [21] effectively predicted tool wear based on a GA–BP neural network model. Liu D et al. [22] compared the accuracy of the BP and GA–BP neural network models for soil water prediction. The test results showed that the GA–BP model could be used to predict soil water for ecological protection. However, the simulation parameters for maize straw calibration using a combination of the discrete element method, physical experiments, and machine learning have not been consulted.

This article used a combination of physical experiments, virtual simulation, and machine learning to calibrate the simulation parameters of maize straw. A bimodal-distribution discrete element model of maize straw was established based on the intrinsic and contact parameters via physical experiments. Taking the relative error of the peak compression force from physical tests and simulations as the test index, the Plackett–Burman test, steepest-climb test, and central composite design test were successively carried out. The results of the central composite design experiment were used as the dataset. GA–BP was used for cycle iteration, and the model’s number of cycle iterations was set. After the iteration was completed, the selection was stopped, and the individual with the closest fitness was obtained. The accuracy of the GA–BP prediction model was verified via physical experimentation. The results provide a theoretical basis for analyzing the damage mechanism of maize straw during the grinding process.

## 2. Materials and Methods

### 2.1. Physical Test of Radial Compression of Maize Straw

**Figure 1:**
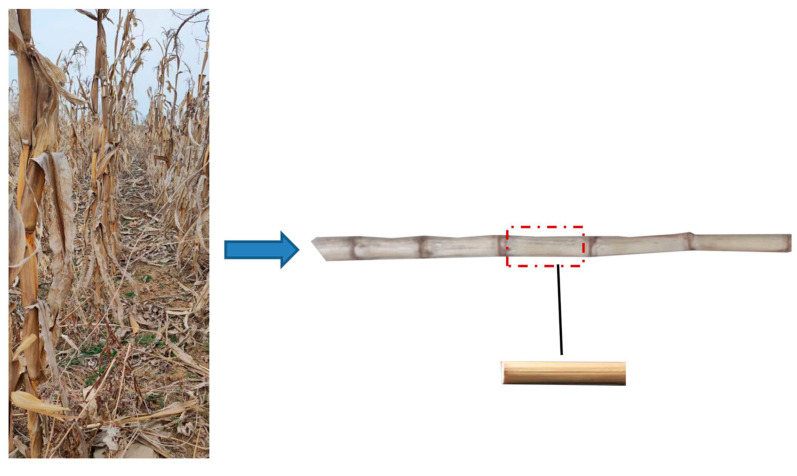
The maize straw.

Natural air-dried maize straw harvested in Xujiazhuang Village, Zhoucun District, Zibo City, was used to make the experimental materials. As depicted in Figure 1, the variety was NK 815. During the experiment, 10 stalks were randomly selected, and the leaf sheaths, bracts, and roots of the corn stalks were removed. The third section of maize straw from the ground was selected. As shown in Table 1, the length and diameter of the maize straw were 90 ± 4.92 mm and 23.78 ± 3.21 mm, respectively. The average moisture content of the maize straw was 8.34%. All the maize straw was transferred into a measuring cylinder, and the volume of discharged water indicated the volume of the maize straw. The quality of the maize straw was measured with a balance. The density was 136.4 kg/m^3^.

**Figure 2:**
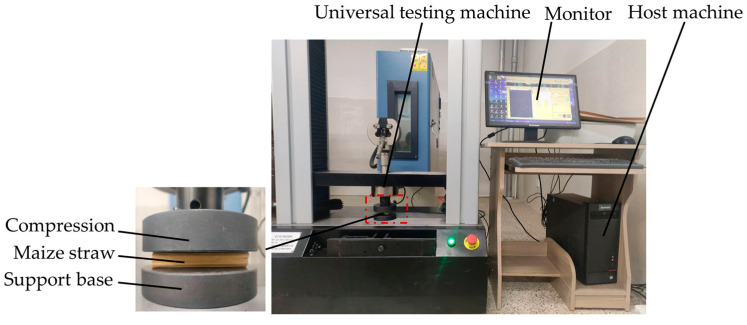
Physical experiment on radial compression of the maize straw.

A radial compression test of the maize straw was conducted using a universal testing machine, as shown in Figure 2. A corn straw with a length of 90 mm was selected and placed horizontally in the center of the support base. The experimental loading speed was 20 mm/min. The experiment was stopped after the maize straw was visibly crushed. After repeating the process 10 times, the peak compression force of the maize straw was 1798 N.

### 2.2. Measurement of Contact Mechanics Parameters

#### 2.2.1. Static Friction Coefficient Measurement

**Figure 3:**
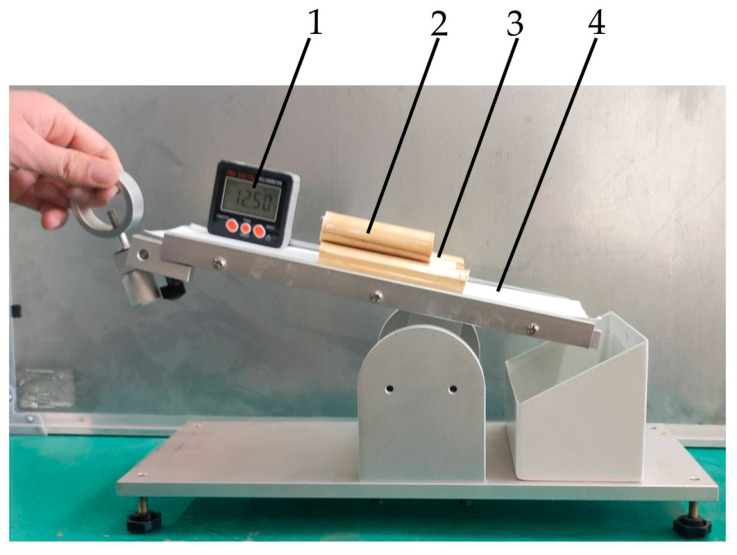
Static friction coefficient measurement device. 1. Digital display inclinometer; 2. tested maize straw; 3. maize straw base plate; 4. test plane.

The static friction coefficients of the maize straw–maize straw and the maize straw–steel plate were measured with an inclined-plane instrument [23]. Before the experiment, the maize straws were neatly and tightly bonded together to form a straw bottom plate. Each plate was placed on the inclined-plane instrument on a horizontal test bench and stuck to the testing plane of the inclined-plane tester. The angle of the test plane was adjusted until the maize straw was observed to slide. The angle on the digital display inclinometer was recorded, as shown in Figure 3. The static friction coefficient between the maize stalks was calculated using Equation (1). Each group of tests was repeated 10 times, taking the average value as the final value. The static friction coefficients of the maize straw–maize straw and the maize straw–steel plate were 0.28 ± 0.05 and 0.45 ± 0.08, respectively.

(1) $$\mu_{1}=\tan\varphi_{1}$$

Here, $\mu_{1}$ is the static friction coefficient, and $\varphi_{1}$ is the critical angle of the static friction coefficient, °.

#### 2.2.2. Rolling Friction Coefficient Measurement

The instruments and testing methods used in the measurement experiment of the rolling friction coefficient were similar to those used for the static friction coefficient [24,25]. The angle of the test plane was adjusted until maize straw rolling was observed. The angle on the digital display inclinometer was recorded. Each group of tests was repeated 10 times, taking the average value as the final value. The rolling friction coefficients of the maize straw–maize straw and the maize straw–steel plate were 0.16 ± 0.15 and 0.24 ± 0.24, respectively.

### 2.3. Establishment of Discrete Element Simulation Model

#### 2.3.1. Hertz–Mindlin with Bonding Contact Model

The Hertz–Mindlin with bonding contact model [26] was used to bond the particles with a finite size in the maize straw model. A bond could resist tangential and normal displacement until the maximum normal and tangential shear stress was reached, and the bond broke. The Hertz–Mindlin contact model was used to calculate the interaction between particles before bond formation. After the bond was generated at a certain time, the bond force and torque were set to zero, and the superimposed increment in the bond force and torque was found in each time step. The normal and tangential strains were calculated using Equation (2):

(2) $$\left\{\begin{array}{l} F_{n}=-\int v_{n}S_{n}A\delta_{t}\\ F_{t}=-\int v_{t}S_{t}A\delta_{t}\\ M_{n}=-\int \omega_{n}S_{t}J\delta_{t}\\ M_{t}=-\int \omega_{t}S_{n}\frac{J}{2}\delta_{t} \end{array}\right.$$

where $A=\pi {R_{B}}^{2}$ is the contact area between particles (mm^2^); $F_{n}$ is the normal force acting on the particles (N); $F_{t}$ is the tangential force acting on the particles (N); $M_{n}$ is the normal moment of the particle (N∙m); $M_{t}$ is the tangential moment of the particles (N∙m); $S_{n}$ is the normal bonding stiffness (N/m^3^); $S_{t}$ is the tangential bonding stiffness (N/m^3^); $v_{n}$ is the normal velocity of particle motion (m/s); $v_{t}$ is the tangential velocity of the particles (m/s); $\omega_{n}$ is the normal relative velocity of the particles (rad/s); $\omega_{t}$ is the tangential relative velocity of the particles (rad/s); $\delta_{t}$ is the time step size (s); $J=0.5\pi {R_{B}}^{4}$ is the moment of inertia of the particles (kg∙m^2^); and $R_{B}$ is the contact radius of the particles (mm).

The bonding force of the maize straw particles mainly depends on five parameters: the normal stiffness coefficient, the tangential stiffness coefficient, the critical normal stress, the critical tangential stress, and the bonding radius. To simplify the simulation calculation, the normal stiffness coefficient of the maize straw particles was equal to the tangential stiffness coefficient, and the critical normal stress was equal to the critical tangential stress. When the normal and tangential stresses on the bond were greater than the set values of the normal or tangential ultimate stress, the bond broke. The calculation formulas for the normal ultimate stress and tangential ultimate stress are shown in Equation (3). The initial range was obtained via the simulation of several maize straw particles’ radial compression, which was the basis for the subsequent simulation test.

(3) $$\left\{\begin{array}{l} \sigma_{\max}<\frac{-F_{n}}{A_{b}}+\frac{2M_{t}}{J}R_{B}\\ \tau_{\max}<\frac{-F_{t}}{A}+\frac{M_{n}}{J}R_{B} \end{array}\right.$$

#### 2.3.2. Establishment of Simulation Model for Radial Compression of Maize Straw

**Figure 4:**
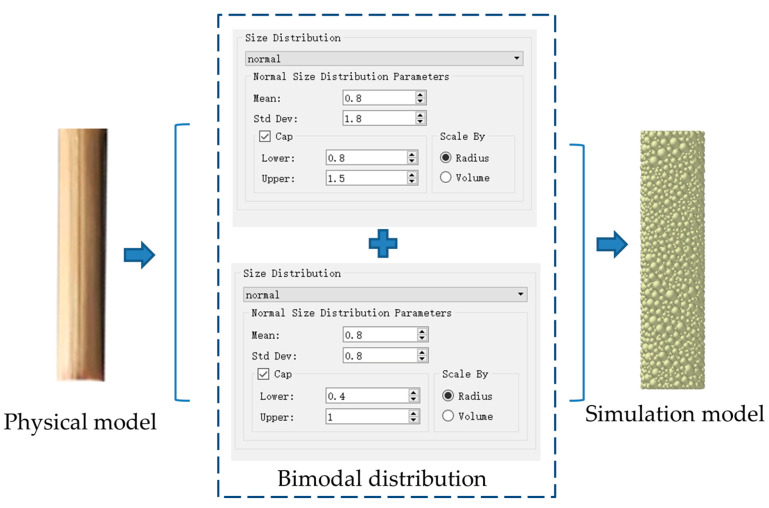
Establishment of the simulation model for maize straw.

In the modeling process, the maize straw was equivalent to an isotropic structure [27,28]. A geometric model of the maize straw particles was created using the Solidworks 2022. The maize straw model was converted into .* stp format and then imported into EDEM 2018. The bimodal-distribution stacking method was used to build the maize straw model, as shown in Figure 4. Spherical particles were selected as the base particles of the maize straw model. The particle radius was set to 1.5 mm. The total number of particles was 3534, among which the large particles occupied the main spatial position, the small particles were closely arranged around the large particles, and the particles had a high coordination number. The adhesive force was stronger, which made the mechanical properties of the particle groups more similar to the actual situation and reduced the computer simulation load [29].

**Figure 5:**
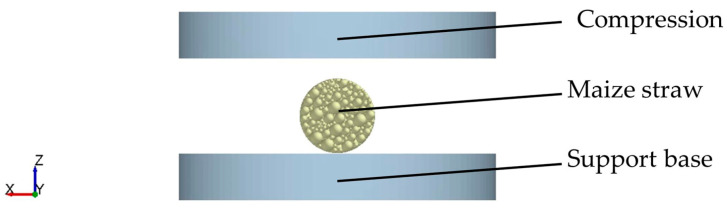
Establishment of a simulation model for maize straw compression.

As shown in Figure 5, the Hertz–Mindlin model (no slip) was selected as the contact model between the maize straw and the compression and support base. The simulated loading speed was 20 mm/min for the maize straw compression. The experiment was stopped after the maize straw was visibly crushed. The Rayleigh time step was set to 15%, the data saving interval was 0.01 s, and the grid size was three times the minimum particle radius.

### 2.4. Calibration of Simulation Parameters for Maize Straw

The Plackett–Burman test was used to analyze the significance of the simulation parameters. The optimal range of the significance parameters was determined using the steepest-climb test. The central composite design was carried out, taking the relative error between the peak compression force of the simulation test and that of the physical test as the experimental indicator. A GA–BP neural network prediction model for the peak compression force of maize straw was established using the experimental data of the central composite design as the dataset. The GA–BP prediction model was analyzed and evaluated. The accuracy of the GA–BP prediction model was verified via physical experiments. Finally, the optimal combination of simulation parameters for the maize straw was obtained.

#### 2.4.1. The Plackett–Burman Test

The parameter range of the Plackett–Burman test was based on the physical experiment results. The other simulation parameters were found in the relevant literature [30,31,32,33,34]. The relative error between the peak compression force of the simulation test and that of the physical test was taken as the test index. The parameters that significantly influenced the response value were selected via the Plackett–Burman test. The minimum and maximum values of the test parameters in Table 2 are coded as −1 and +1, representing the low and high parameter levels, respectively.

#### 2.4.2. Steepest-Climb Test

The Plackett–Burman test was used to analyze the significance of the simulation parameters. Then, the optimal range of the significance parameters was determined by the steepest-climb test. All other non-significant parameters were averaged. The relative error between the peak compression force of the simulation test and that of the physical test is shown in Equation (4):

(4) $$P=\left|\frac{F-F_{f}}{F}\right|\times 100\%$$

where *P* is the relative error between the peak compression force of the simulation test and that of the physical test (%); *F* is the peak compression force of the simulation test (N); and *F_f_* is the peak compression force of the physical test (N).

### 2.5. Regression Fitting Modeling Based on Machine Learning Algorithms

#### 2.5.1. Principle of BP Neural Network

**Figure 6:**
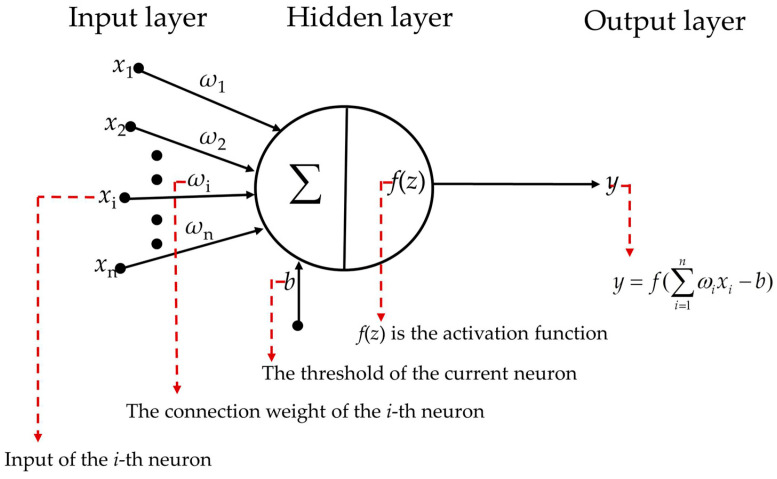
Neuron model.

The BP (backpropagation) neural network is a multi-layer feedforward neural network trained using the error backpropagation algorithm, mainly composed of three layers: the input layer, hidden layer, and output layer [35,36]. Neurons connect each layer to another, transmitting information between them. The gradient descent method adjusts the weight and threshold of the neural network via error backpropagation to minimize the network error. As shown in Figure 6, the input (*x*_n_) of each neuron is multiplied by their respective weights (*ω*_n_) and then subtracted from the bias vector (*b*). The sum of the inputs is passed through an activation function to obtain a specific neuron output (*y*).

#### 2.5.2. Sample Construction

The optimal range of the significant influencing factors was obtained via the Plackett–Burman test and the steepest-climb test. The central composite design was carried out by taking the relative error between the peak compression force of the simulation test and that of the physical test as the experimental indicator. The horizontal coding of the simulation parameters for maize straw is shown in Table 3.

#### 2.5.3. BP Neural Network Construction and Training

**Figure 7:**
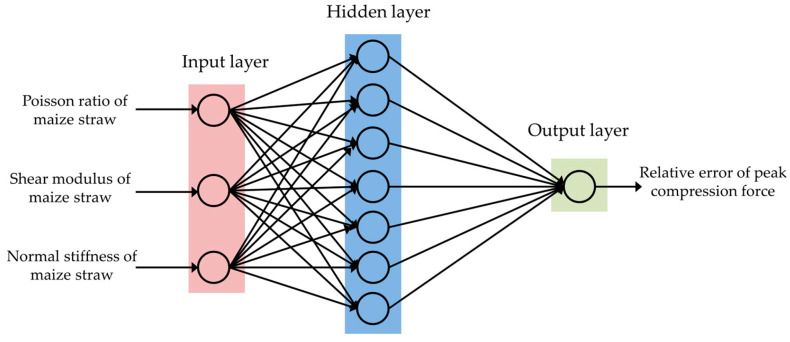
Neural network topology.

In the construction process of the BP neural network, the number of input layer nodes was determined by the number of input parameters. The number of the output layer nodes was determined by the number of output parameters. Multiple hidden layers are often added between the input and output layers. When the number of hidden layers increases, the accuracy increases, but the network structure is complex, and the learning efficiency decreases. When the input node of the BP artificial neural network is n, the number of hidden layer nodes in the network is selected as 2n + 1 [37]. This article adopted a single hidden layer structure, with 7 hidden layer nodes selected. The BP neural network model adopted a three-layer network, with a neural network structure of 3-7-1. The network topology is shown in Figure 7.

The results of the central composite design experiment were used as the dataset. The experimental design and results are shown in Table 4. The total data (21 groups) were randomly divided into 17 groups (80%) for training, and 6 groups (20%) for testing and verification to avoid over-training and over-parameterization. In the training process, the transfer function from the input layer to the hidden layer was selected as the sigmoid function. The nonlinear damped least-square (LM) optimization algorithm was used in the training algorithm. The mapminmax function was selected to normalize the input and output data to eliminate dimensional effects.

#### 2.5.4. Optimization of the BP Neural Network Model with Genetic Algorithm (GA–BP)

**Figure 8:**
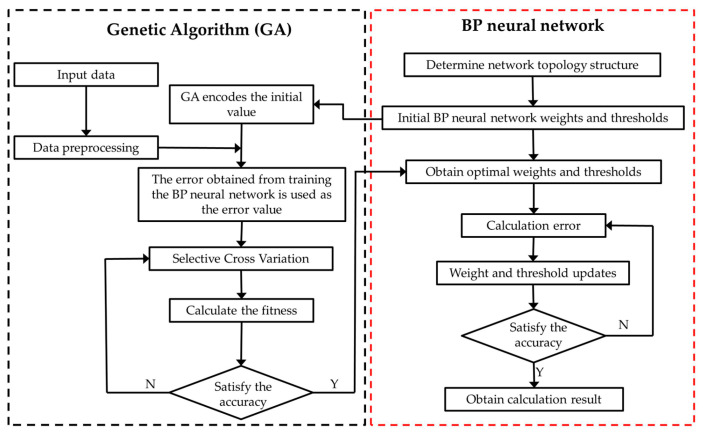
The training process of GA–BP optimization algorithm.

The genetic algorithm was used to optimize the initial weight and threshold of the BP neural network model, improve its computational efficiency and prediction accuracy, and build the collaborative mechanism of the genetic algorithm and BP neural network model. The GA–BP neural network is a hybrid algorithm combining the genetic algorithm and error backpropagation algorithm to train a feedforward artificial neural network, which can accelerate the convergence speed and avoid local minimization. This network converges quickly and easily reaches the optimal solution. The process of the GA–BP optimization algorithm involved using the genetic algorithm to optimize the initial weights and thresholds of the BP network before executing the BP algorithm. After genetic completion, the optimized initial weights and thresholds were assigned to the BP neural network for updating learning so that the optimized BP neural network could better predict the output of the function, as shown in Figure 8.

#### 2.5.5. Data Analysis and Processing

Matlab R2022b software was used as the algorithm-running platform. The predictive performance of the machine learning model was evaluated by the R^2^, RMSE, and MAE. The larger the R^2^, the higher the model fit. The lower the RMSE and MAE, the better the model’s accuracy and stability. GA–BP was used for cycle iteration, and the number of cycle iterations of the model was set. After the iteration was completed, the selection was stopped and the individual with the closest fitness was obtained. The accuracy of the model was verified via physical experiments.

## 3. Results and Analysis

### 3.1. Analysis of Plackett–Burman Test Results

Since many factors affected the compression test between the maize straw and steel plate, the Plackett–Burman test was needed to determine the significance of each factor’s influence on the radial compression test. The contact parameters between the maize straw and steel plate were screened with the peak compressive force as the response value. A total of 12 groups of tests were carried out. Each group of tests was repeated three times, and the average value was taken. The experimental design and results are shown in Table 5. The significance analysis of the Plackett–Burman test results is shown in Table 6. The significance analysis revealed that X_1_, X_2_, and X_5_ had a significant impact on the peak compression force of the maize straw and steel plate. Therefore, further analysis of the influence law of the X_1_, X_2_, and X_5_ factors on the peak compressive force was needed.

The design scheme and results of the steepest-climb test are shown in Table 7. The peak compression force increased with the increase in the Poisson ratio, shear modulus, and normal stiffness coefficient of the maize straw. The relative error first increased and then decreased. The relative error of the third group was the smallest, and the central point of the central composite design test was selected. The optimal range intervals for X_1_, X_2_, and X_5_ were determined to be 0.32–0.36, 1.24 × 10^8^–1.72 × 10^8^ Pa, and 5.9 × 10^6^–6.7 × 10^6^ N/m^3^, respectively.

### 3.2. Regression Model Based on Machine Learning

#### 3.2.1. GA–BP Model Training Results

**Figure 9:**
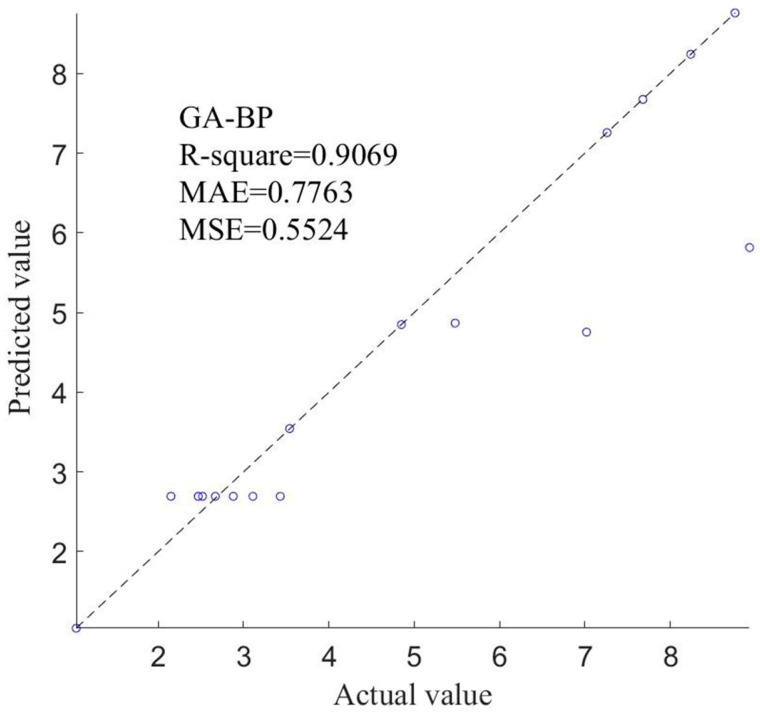
The variation pattern between measured and predicted values.

The change law of the measured and predicted values of the GA–BP is shown in Figure 9. The evaluation index R^2^ was 0.9069, the RMSE was 0.5524, and the MAE was 0.7763, which showed a good performance in model accuracy, stability, and fitting. This shows that the GA–BP algorithm achieved a better fitting effect in this study and constructed the model with higher precision and less error. This model could be used for further analysis.

#### 3.2.2. Model Evaluation

**Figure 10:**
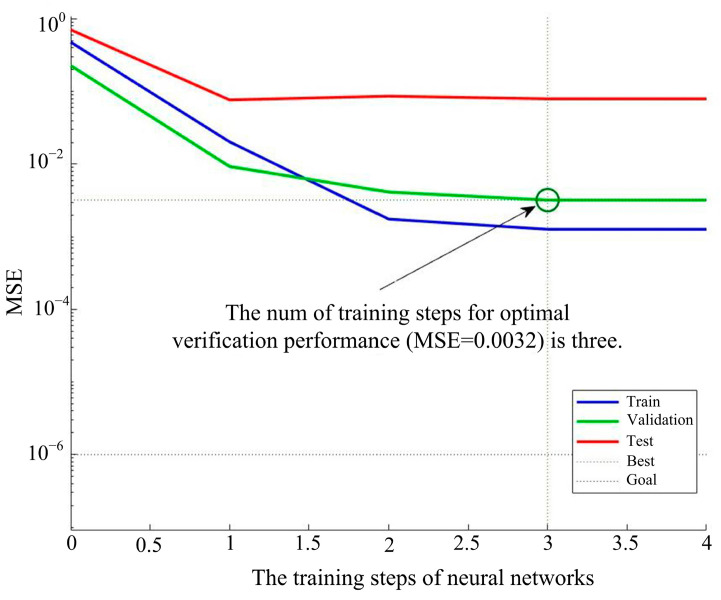
The MSE performance evaluation of the model.

The MSE performance evaluation of the GA–BP algorithm model was carried out, as shown in Figure 10. The MSE of the model showed a decreasing trend during the training process. The fitting effect of the model to the training data gradually improved as the training progressed. The best performance was obtained at the third step of training, which indicates that the GA–BP model’s training convergence is fast and stable.

**Figure 11:**
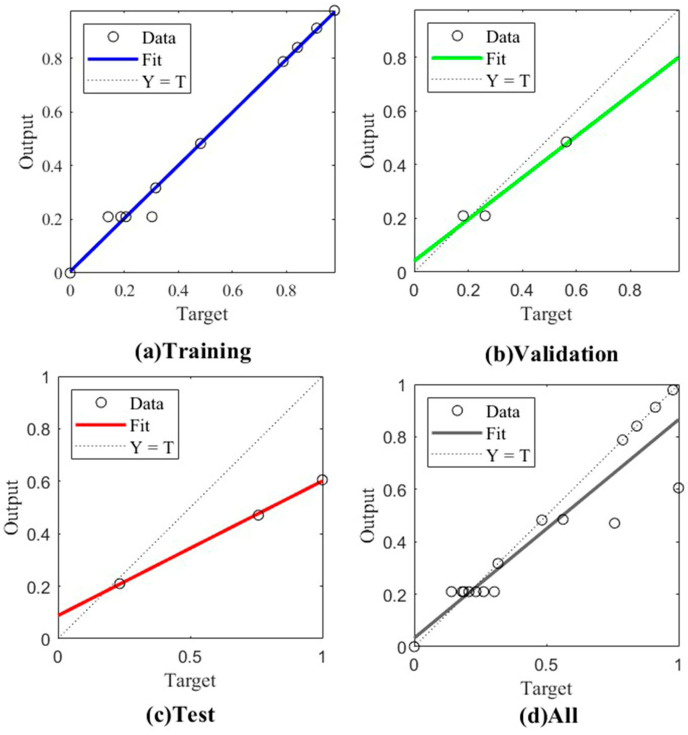
Regression analysis.

The training, validation, testing, and comprehensive performance changes of the GA–BP are shown in Figure 11. The correlation coefficients for the training, validation, testing, and comprehensive data were 0.9930, 0.9994, 0.9989, and 0.9386. This indicates that the model had a strong fitting effect and good generalization ability. The correlation coefficients of the data were very close, indicating no obvious overfitting or underfitting. The GA–BP algorithm was used to obtain a model with high precision and strong generalization ability, which could be used for subsequent experimental research.

#### 3.2.3. GA–BP Optimization Test

The GA–BP algorithm was used for cycle iteration, and the number of cycle iterations of the model was set to 150 times. After the iteration was completed, the selection was stopped, and the individual with the closest fitness was obtained. The results show that the Poisson ratio, shear modulus, and normal stiffness of the maize straw were 0.357, 1.511 × 10^8^ Pa, and 6.285 × 10^6^ N/m^3^, respectively. The peak compression force simulation experiment of the maize straw was conducted using the GA–BP algorithm optimized parameter combination, and the relative error was 1.14%. The established model can be used for the discrete element simulation of maize straw crushing.

### 3.3. Discussion

The intrinsic parameters and contact parameters of the maize straw were measured via physical tests. The static friction coefficients of the maize straw–maize straw and the maize straw–steel plate were 0.28 and 0.45, respectively. The rolling friction coefficients of the maize straw–maize straw and the maize straw–steel plate were 0.16 and 0.24, respectively. The other simulation parameters can be found in the relevant literature. The significance analysis of the simulation parameters was conducted using the Plackett–Burman experiment. It was found that the Poisson ratio, shear modulus, and normal stiffness of the maize straw significantly impacted the peak compression force of the maize straw and steel plate. The steepest-climb test was carried out for the significance parameter, and the relative error between the peak compression force in the simulation test and that in the physical test was used as the evaluation index. The optimal range intervals for the Poisson ratio, shear modulus, and normal stiffness of the maize straw were determined to be 0.32–0.36, 1.24 × 10^8^–1.72 × 10^8^ Pa, and 5.9 × 10^6^–6.7 × 10^6^ N/m^3^, respectively. A GA–BP algorithm neural network prediction model for the peak compression force of the maize straw was established using the experimental data of the central composite design as the dataset and was analyzed and evaluated. The accuracy of the GA–BP algorithm prediction model was verified via experiments. The ideal combination of parameters was found to be 0.357 for the Poisson ratio, 1.511 × 10^8^ Pa for the shear modulus, and 6.285 × 10^6^ N/m^3^ for the normal stiffness of the maize straw.

In this paper, the Rayleigh time step was set to 15%, which was slightly high for a simulation including breakage. We will change the Rayleigh time step, particle size, and particle ratio to carry out further research.

## 4. Conclusions

(1) The intrinsic parameters and contact parameters of the maize straw were measured via physical tests. The static friction coefficients of the maize straw–maize straw and the maize straw–steel plate were 0.28 and 0.45, respectively. The rolling friction coefficients of the maize straw–maize straw and the maize straw–steel plate were 0.16 and 0.24, respectively. Other simulation parameters can be found in the relevant literature.

(2) The significance analysis of the simulation parameters was conducted via the Plackett–Burman experiment. It was found that the Poisson ratio, shear modulus, and normal stiffness of the maize straw significantly impacted the peak compression force of the maize straw and steel plate. The steepest-climb test was carried out for the significance parameter, and the relative error between the peak compression force in the simulation test and that in the physical test was used as the evaluation index. The optimal range intervals for the Poisson ratio, shear modulus, and normal stiffness of the maize straw were determined to be 0.32–0.36, 1.24 × 10^8^–1.72 × 10^8^ Pa, and 5.9 × 10^6^–6.7 × 10^6^ N/m^3^, respectively.

(3) A GA–BP algorithm neural network prediction model for the peak compression force of the maize straw was established using the experimental data of the central composite design as the dataset and was analyzed and evaluated. The accuracy of the GA–BP algorithm prediction model was verified via experiments. The ideal combination of parameters was 0.357 for the Poisson ratio, 1.511 × 10^8^ Pa for the shear modulus, and 6.285 × 10^6^ N/m^3^ for the normal stiffness of the maize straw.

## Figures and Tables

**Table 1:** Size parameters of the maize straw.

Maize Straw	1	2	3	4	5	6	7	8	9	10	Average Value (mm)
Length of maize straw (mm)	84	87	86	91	86	87	98	99	93	89	90
Length of maize straw (mm)	23.64	20.82	19.48	20.04	22.22	26.47	28.16	29.29	25.02	22.66	23.78

**Table 2:** Plackett–Burman test parameter table.

No.	Test Parameter	Code		
		−1	0	+1
X_1_	Poisson’s ratio of maize straw	0.3	0.35	0.4
X_2_	Shear modulus of maize straw (Pa)	1 × 10^8^	1.6 × 10^8^	2.2 × 10^8^
X_3_	Collision recovery coefficient between maize straw	0.34	0.47	0.60
X_4_	Collision recovery coefficient between maize straw and steel plate	0.38	0.52	0.66
X_5_	Normal stiffness coefficient (N/m^3^)	5.5 × 10^6^	6.5 × 10^6^	7.5 × 10^6^
X_6_	Tangential stiffness coefficient (N/m^3^)	5.5 × 10^6^	6.5 × 10^6^	7.5 × 10^6^
X_7_	Normal critical stress (MPa)	4.5	5.0	5.5
X_8_	Tangential critical stress (MPa)	5.2	6.0	6.8
X_9_	Bonding radius (mm)	1.8	2.0	2.2

**Table 3:** The horizontal coding table of the simulation parameters.

Level	Parameter		
	X_1_	X_2_	X_5_
−1.682	0.293	1.413 × 10^8^	6.233 × 10^6^
−1	0.32	1.44 × 10^8^	6.26 × 10^6^
0	0.34	1.48 × 10^8^	6.30 × 10^6^
+1	0.36	1.52 × 10^8^	6.34 × 10^6^
+1.682	0.387	1.547 × 10^8^	6.367 × 10^6^

**Table 4:** Experimental design and results.

No.	X_1_	X_2_	X_5_	Relative Error P (%)
1	0	0	0	1.82
2	1	1	−1	1.04
3	0	0	−1.682	4.85
4	1.682	0	0	4.4
5	0	0	0	2.47
6	−1	−1	−1	8.76
7	0	0	0	2.52
8	0	0	0	1.96
9	−1	−1	1	8.93
10	0	0	1.682	7.15
11	−1	1	1	6.41
12	−1.682	0	0	8.24
13	1	1	1	7.68
14	1	−1	−1	2.39
15	0	0	0	2.88
16	−1	1	−1	5.48
17	0	0	0	3.43
18	0	0	0	2.15
19	0	0	0	2.67
20	0	−1.682	0	7.02
21	0	0	0	3.11
22	1	−1	1	7.26
23	0	1.682	0	3.54

**Table 5:** The Plackett–Burman experimental design and results.

No.	Parameter									Relative Error P (%)
	X_1_	X_2_	X_3_	X_4_	X_5_	X_6_	X_7_	X_8_	X_9_	
1	1	−1	−1	−1	1	−1	1	1	−1	9.14
2	1	−1	1	1	1	−1	−1	−1	1	22.24
3	−1	1	1	1	−1	−1	−1	1	−1	4.49
4	−1	−1	−1	−1	−1	−1	−1	−1	−1	10.29
5	1	1	−1	−1	−1	1	−1	1	1	28.41
6	1	1	1	−1	−1	−1	1	−1	1	8.64
7	1	−1	1	1	−1	1	1	1	−1	25.42
8	−1	1	1	−1	1	1	1	−1	−1	15.5
9	−1	1	−1	1	1	−1	1	1	1	1.33
10	−1	−1	−1	1	−1	1	1	−1	1	3.52
11	−1	−1	1	−1	1	1	−1	1	1	6.66
12	1	1	−1	1	1	1	−1	−1	−1	25.29

**Table 6:** Significance analysis of the Plackett–Burman test.

Parameter	Effect	Mean Square Sum	Impact Rate	Significance Order
X_1_	201.083	121,304	12.28	2
X_2_	453.217	616,216	62.37	1
X_3_	42.9167	5525.52	0.56	9
X_4_	99.6167	29,770.4	3.01	6
X_5_	−190.383	108,737	11.01	3
X_6_	63.75	12,192.2	1.23	7
X_7_	121.35	44,177.5	4.47	4
X_8_	45.2833	6151.74	0.62	8
X_9_	101.517	30,916.9	3.13	5

**Table 7:** Analysis of steepest-climb test results.

No.	Parameter			Peak Compression Force F (N)	Relative Error P (%)
	X_1_	X_2_	X_5_		
1	0.3	1.0 × 10^8^	5.5 × 10^6^	1437.71	25.06%
2	0.32	1.24 × 10^8^	5.9 × 10^6^	1653.79	8.72%
3	0.34	1.48 × 10^8^	6.3 × 10^6^	1752.78	2.58%
4	0.36	1.72 × 10^8^	6.7 × 10^6^	1744.61	3.06%
5	0.38	1.96 × 10^8^	7.1 × 10^6^	2076.45	13.41%
6	0.4	2.2 × 10^8^	7.5 × 10^6^	2588.91	30.55%

## Data Availability

The data presented in this study are available upon request from the corresponding author.
